# The Efficacy of Conventionally Fractionated Radiation in the Management of Osseous Metastases from Metastatic Renal Cell Carcinoma

**DOI:** 10.1155/2018/6384253

**Published:** 2018-01-09

**Authors:** Rohit Gunan Ganju, Mindi TenNapel, Nicholas Mahan, Amir Zahra, Xinglei Shen

**Affiliations:** Department of Radiation Oncology, University of Kansas Cancer Center, Kansas University Medical Center, Kansas City, KS, USA

## Abstract

**Background:**

There is little data regarding the effectiveness of palliative radiation with conventional fractionation for metastatic renal cell carcinoma (RCC), which has been described as radioresistant. We conducted a retrospective analysis of patients with metastatic bony disease from RCC treated with radiation therapy at our institution.

**Methods:**

Forty patients with histologically confirmed RCC with a total of 53 treatment courses were included. Pain response after radiotherapy was recorded and freedom from progression was generated using posttreatment radiographs. Patient data was analyzed to assess influence on local control.

**Results:**

Patients had a median age of 63. Median follow-up was 9.3 months. The most common radiation dose was 30 Gy in 10 fractions. Pain control after radiotherapy was achieved in 73.6% of patients. Increasing age was associated with nonresponse at the initial pain assessment post-RT (*p* = 0.02). In lesions with initial pain response, nonclear cell histology was associated with increased pain recurrence (*p* = 0.01) and a shorter duration to pain recurrence (*p* = 0.01). Radiographic control at 1 year was 62%.

**Conclusions:**

Pain response and control rates for osseous metastatic disease in RCC are comparable to other histologies when treated with conventional fractionation. These appear to be inferior to reported control rates from stereotactic treatments.

## 1. Introduction

The incidence of renal cell carcinoma (RCC) has been increasing in the developing world and accounts for 2-3% of all new cancer diagnoses worldwide [1, 2]. Within the US, 62,700 cases were diagnosed in 2016. Increased use of cross-sectional imaging has resulted in more patients diagnosed in earlier stages. However, up to 30% of patients present with metastatic disease, and 25% of patients will develop metastatic disease following definitive treatment for nonmetastatic RCC [3]. Recent advances have led to the development of targeted therapies, and 12% of patients with distant disease survive 5 years or more [4, 5]. Given this, symptomatic palliation plays a large role in management of this disease.

Skeletal metastases play a major role in the quality of life of patients with metastatic RCC. A recent analysis of the Nation Wide Inpatient Sample showed that 144,899 US hospital admissions from 1998 to 2010, accounting for 20.8% of hospital visits for patients with RCC, were due to skeletal related events [4]. In a study of patients with solid tumor metastases to bone, patients with RCC treated with placebo suffered from more skeletal related events compared to other patient groups [6]. As a result, effective palliation of bone metastases remains an important aspect in the management of RCC.

Bone metastases from RCC have previously been described as radioresistant with limited duration of response after treatment [7]. Previous retrospective studies have shown improved response associated with higher doses of radiotherapy in patients with metastatic disease [8, 9]. More recently, there has been increasing utilization of stereotactic body radiation therapy (SBRT) in the treatment of bone metastasis from RCC to overcome their radioresistant nature [10–16]. However, the data is conflicting, with some studies showing no benefit to increased dose, especially in the modern era of targeted RCC therapies [17]. In an effort to better understand factors associated with a positive response to therapy, we conducted a retrospective analysis of patients treated for RCC and metastatic bone disease with conventional radiation therapy at our institution.

## 2. Materials and Methods

### 2.1. Patients

After obtaining Institutional Review Board approval, records were obtained for patients treated with radiation therapy for bone metastasis from RCC at our institution from 2009 to 2016. We identified 40 patients who underwent 53 treatment courses of radiation therapy. All patients completed a palliative course of radiation therapy (RT) as prescribed by the treating radiation oncologist. All patients had at least one month of follow-up. Of the cases identified, we collected patient age, sex, treatment site, surgical intervention, histology (clear cell or nonclear cell), initial pain response post-RT, and evidence of progression. Evidence of progression was captured by radiographic analysis for all patients and by self-report in patients who had a partial or complete initial pain response post-RT. Treatment sites for bone metastasis were recorded and analyzed as vertebrae, long bones, pelvis, and others (ribs, clavicle, and clivus).

### 2.2. Treatment

Previous systemic treatment for metastatic disease was recorded and included single or multiple drug therapies. The number of prior lines of systemic therapy at the time of radiotherapy were recorded. Systemic treatment recorded included tyrosine kinase inhibitors (TKIs), mammalian target of rapamycin (mTOR) inhibitors, cytokine therapy (IL-2), monoclonal antibodies, and antimetabolites. Surgical intervention was recorded as the presence of surgery at the treatment site prior to radiation therapy.

Radiation was prescribed to bony sites for palliation of pain or postoperatively to prevent local recurrence. Radiation treatment courses were recorded as total dose in Gray (Gy) and number of treatment fractions delivered. Prescribed treatment dose and fractionation were at the discretion of the treating physician and were based on clinical factors and physician preference. Actual, rather than prescribed, treatment courses were recorded as there were occasional changes in dose prescription midtreatment due to patient refusal or toxicity. Radiation treatment courses ranged from 8 to 45 Gy in 1 to 15 fractionated treatments to metastatic sites. Multiple different fractionation treatment regimens were used, and biological effective dose (BED) was calculated for each treatment. BED was calculated using an *α*/*β* ratio of 7, as has been commonly used in previous studies [10, 11, 17]. The most common treatment dose prescribed was 30 Gy in 10 fractions.

### 2.3. Response Assessment

Patients were assessed for both clinical and radiographic response following treatment. Initial pain response was recorded as either complete response (total resolution of symptoms requiring no narcotic use), partial response (improved symptoms), or nonresponse (stable or worse pain after treatment) at the treated site based on patient report at the initial follow-up, which generally occurred one to three months from the end of treatment. Radiographic studies were reviewed for changes after treatment and were recorded as partial response, stable disease, or local progression. Pain progression was defined by an increase in pain from the initial post-RT-treatment response to follow-up responses; this was evaluated subjectively by patient self-report of either increased pain at the treated site or an increase in narcotic requirements to achieve similar pain control at the treated site. Radiographic progression was defined by local progression. Duration was calculated in months from the end of RT treatment to the date of patient follow-up or date of radiographic study.

### 2.4. Statistical Analysis

Logistic regression was used to analyze if patient age, sex, treatment site, BED, histology, presence of surgery, and prior systemic treatment were associated with pain progression or radiographic progression. Initial pain response was analyzed to determine if it was associated with radiographic progression. Kaplan-Meier curves were generated to analyze if patient age, sex, treatment site, BED, histology, presence of surgery, and prior systemic treatment were associated with time to progression. Initial pain response was analyzed to see if it was associated with time to radiographic progression.

As each metastatic site had a separate treatment course and pain assessment, each was analyzed individually. However, if multiple metastasis occurred in a single patient, treatment decisions for one lesion may influence treatment for other lesion(s). To account for this, sensitivity analyses were run on a subset of data where only one lesion for each patient was included to ensure the overall trend of the results was the same. All tests were 2-tailed, and differences of 0.05 were considered statistically significant. Statistical analyses were conducted using the Statistical Analysis System (SAS Institute, Cary, NC.).

## 3. Results

### 3.1. Patient and Treatment Characteristics

Patients treated at our institution for metastatic RCC to bone from 2009 to 2016 were analyzed retrospectively for response to treatment. All patients completed their prescribed radiation treatments and follow-up data from both radiation oncology and medical oncology was analyzed. 40 unique patients who underwent 53 treatment courses with radiotherapy were identified (Table 1). A majority of the patients were male (77.5%). The median age of the patients was 63 years (range 39–82) and the median follow-up was 9.3 months (range 1.6–71.5).

There were 38 lesions treated in 31 men and 15 lesions treated in 9 women; however, this difference was not significant (*p* = 0.1552). There were 29 patients who had a single lesion, 9 patients had 2 lesions, and 2 patients had 3 lesions. In patients with multiple lesions a majority most were diagnosed at the same time. There were 2 patients who had 2 lesions and 1 patient with 3 lesions diagnosed at different time points.

All 53 metastatic lesions treated received the full prescribed course of radiation. As shown in Table 1, there were a number of different sites treated and radiation schemes used. The median BED was 43 (range 17–74). 20 of 53 lesions were treated with surgery and 26 of 53 lesions were treated with concurrent treatment. 44 of the 53 lesions were clear cell histology.

### 3.2. Pain Response

**Figure 1:**
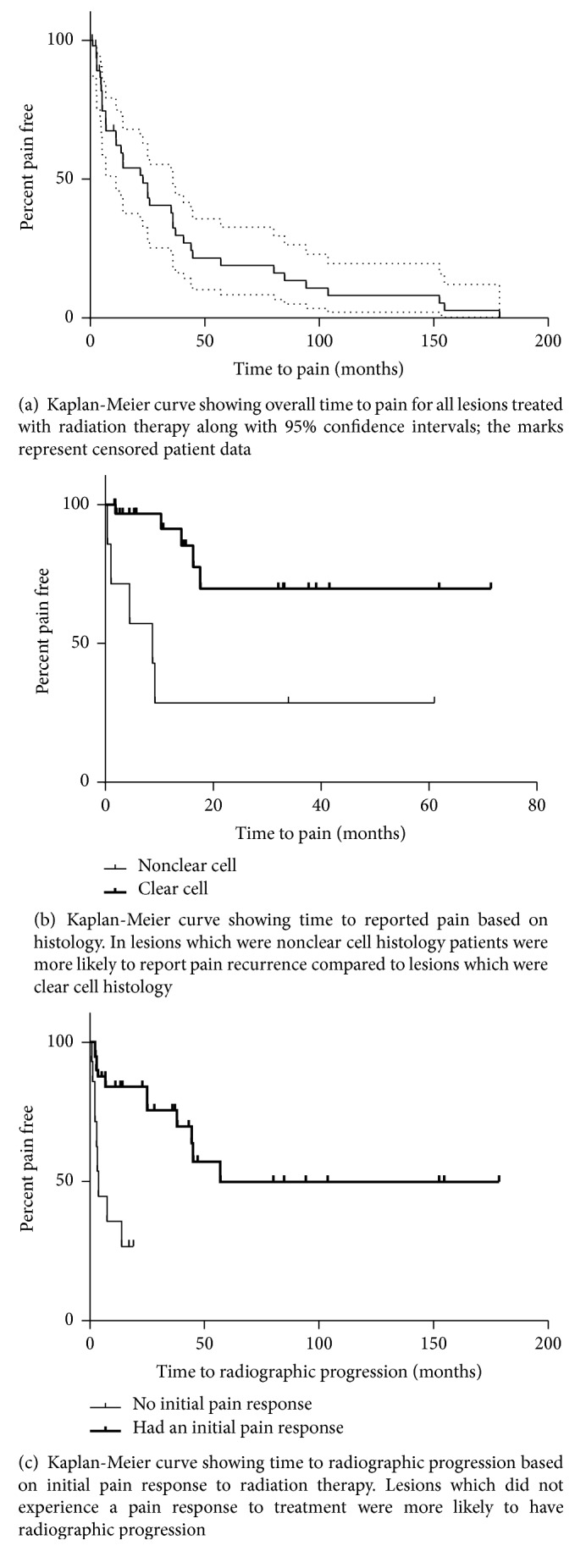


Pain control after radiotherapy was achieved in 39 of 53 (73.6%) of lesions which completed radiotherapy (Figure 1(a)). The median duration of pain response was 22.9 months. 29 of 39 (74.3%) had durable pain control at last follow-up. Durable pain control at 6 months, 1 year, and 2 years were 88.8%, 76.7%, and 61.0%.

Increasing age was associated with nonresponse at initial pain response assessment (*p* = 0.0201; OR = 1.095, 95% CI: 1.014, 1.182). Sensitivity analysis showed a similar trend.

On univariate and multivariate analysis, BED, gender, treatment site, concurrent systemic therapy, and histology (clear cell versus nonclear cell) were not associated with initial pain response (Table 2).

Although histology was not associated with initial pain response, nonclear cell histology had a higher rate of pain recurrence after treatment (Figure 1(b)). In the 39 lesions with a partial or complete initial pain response, 5 of 7 (71%) nonclear cell histology had pain recurrence compared to 5 of 32 (16%) clear cell histology (*p* = 0.0072). Nonclear cell histology was also associated with time to pain recurrence compared to clear cell histology (*p* = 0.007; HR = 5.631, 95% CI 1.618, 19.593). An estimated 71.4% of nonclear cell lesions had pain recurrence at 1 year compared to only 8.7% of clear cell, and only an estimated 30.0% of clear cell had reported a pain recurrence at 2 years.

### 3.3. Local Control

Radiographic control was achieved in 32 of 53 lesions (60.4%) at last follow-up. 6 month, 1 year, and 2 year rates of radiographic control were 69.4%, 62.4%, and 41.3%.

Patient age, sex, treatment site, number of systemic treatment courses prior to radiotherapy, histology (clear cell or not clear cell), and presence of surgery at treatment site were not associated with treatment response.

The only predictor of freedom from local progression was initial pain response (Figure 1(c)). Of the 14 who reported nonresponse at initial pain response assessment, 9 (64%) had radiographic progression compared to a 12 of 39 (31%) who reported a partial or complete initial pain response (*p* = 0.0332). The estimated median time to radiographic progression for nonresponders was 1.5 months compared to 22.8 months in partial or complete responders (*p* < 0.001; HR = 6.732, 95% CI 2.38, 19.05). Sensitivity analysis showed a similar trend.

## 4. Discussion

RCC is described as a less radiosensitive tumor based on in vitro studies and studies of adjuvant radiation therapy in renal cell cancer. Because RCC is perceived to be a less radiosensitive histology, there is increasing use of very high dose radiation using stereotactic body radiation (SBRT) in this population. It is not clear whether SBRT is necessary to achieve adequate control and pain relief. We performed a retrospective analysis of patients treated palliatively for renal cell metastases to bone with conventionally fractionated radiation in the modern era to evaluate response to conventional fractioned radiation. We found 73.6% response in pain control and radiographic response in 60.4% of lesions. The most common treatment course studied was 30 Gy in 10 fractions.

Our results are similar to an older study by Princess Margaret Hospital, which found 83% response in pain with 23 patients treated using 30 Gy in 10 fractions [7]. An additional study from Dibiase et al. also showed greater than 80% pain response to conventionally fractionated radiation doses in 107 patients with renal cell carcinoma, including 89 bone lesions [8].

Despite the evidence that RCC is less radiosensitive than other tumors, these rates are similar to pain response rates expected with conventional radiation therapy for other tumor histologies considered to be more radiosensitive. In the RTOG 9714 trial of palliative radiation for bone metastasis from breast or prostate cancer, the overall pain response rate at 3 months was 66% [18]. The mechanism of pain response may include stromal and inflammatory factors separate from the tumor. Our results support that palliation of pain may be achieved without need for high dose treatments.

There was no significant association found between higher BED and improved pain control in our study. This is consistent with results reported by Wilson et al., who evaluated 143 treatments in 78 patients with metastatic RCC, including 72 bony lesions, treated with conventional fractionation and did not find response rates predicted by BED7 [17].

Amini et al. compared 46 patients with 95 lesions, 45 of which were treated with conventional fractionation and 50 treated with SBRT. Patients who received SBRT had significantly improved symptom control rates. BED > 80 was noted to be correlated with clinical local control [10]. Jhaveri et al. also noted a dose response in their review of their patients, with patients receiving a BED > 85 achieving faster and more durable pain relief than patients receiving BED < 85 [11]. These findings indicate that dose-escalation may play a more significant role in stereotactic treatments.

Other studies have examined the role of SBRT in the management of bone metastases from RCC. These have included treatment to bony metastases as well as spinal lesions, though interpretation of these studies is limited by their retrospective nature. In studies examining exclusively osseous metastases, crude local control rates range from 72 to 100%. A systematic review by Kothari et al. in 2015 looked at 10 studies in which patients with extracranial metastases from renal cell carcinoma were treated with stereotactic radiation, five of which studied patients with bony metastases only [19]. Weighted crude local control rates, as defined by radiographic progression, were 89%, with weighted one-year local control rates being 86%, higher than our reported rates of 60%. Five of these studies reported on pain control, with improvement in pain in 69% of patients, which is comparable to our study. Studies published since this review show comparable local control rates [20–22].

Pain response in our study was correlated with histologic type of tumors, with patients with nonclear cell histology performing significantly worse than those with clear cell histology. Clear cell RCC is the most common subtype of RCC, comprising about 75–90% of total diagnosed RCC. Few studies have examined the effect of histology on local control in extracranial metastatic RCC [2]. In our study, only 2 of 7 patients with nonclear cell histology had durable pain control with treatment. Ghia et al. examined multifraction versus single-fraction treatment for RCC spinal metastasis and examined nonclear cell histology as a predictor for local control [22]. They included 27 patients with clear cell histology and 16 patients without and demonstrated a trend towards worse outcomes in nonclear cell patients, though nonsignificant. The poor response rate in these nonclear cell patients may indicate an inherent radioresistance more so in these patients compared to those with clear cell RCC.

The use of TKIs has improved survival in patients with metastatic RCC. We did not find an association between use of TKI and improved pain response or local control in patients treated with conventional fractionated radiation. This contrasts with a recent study by Miller et al. who registered improved local control in patients receiving SRS for spinal metastasis in systemic-therapy naïve patients [23]. These disparities may be accounted for by the known radiobiological differences between conventional and stereotactic radiation. In addition, this effect appears to be enhanced in patients who are systemic therapy naïve, which was not consistent with our population [23, 24].

The only variable that predicted for radiographic control in our population was initial pain response. The pathophysiology of pain from bone metastases is multifactorial, and response to treatment may be a result of multiple processes [25]. Since the pain response is often seen prior to the first repeat radiograph, early pain response may serve as a surrogate for tumor response or radiosensitivity in these patients.

The current study is limited by its retrospective nature, short-term follow-up, and variability in treatment, with numerous different dosage, fractionation, and lines of systemic therapy being used in treated patients. Additionally, treatment pain response was patient reported and was subject to multiple cofounders. Patients with multiple metastases may have had changing narcotic requirements as a result of their untreated metastases, which was not captured by our data. As a result, we relied on patient self-report of pain recurrence rather than more objective measures. We used BED7 to standardize differences amongst radiation fractionation schemes. However, the use of BED7 is controversial as well, as previous studies have used *α*/*β* ratios of 3, 7, and 10; this may have affected our results. The small number of patients may have limited our ability to detect true differences between subgroups. Lastly, each lesion was analyzed independently; this may mask patient-specific factors that otherwise would account for patient response.

The role of systemic therapy in the management of metastatic renal cell carcinoma is a rapidly evolving one, particularly with the emergence of anti-PD-1 and anti-PD-L1 inhibitors as effective treatment options [26]. The interplay of radiation therapy and these agents is an area yet relatively unexplored [27], though one that is being researched heavily, with the thought that radiation may potentiate the response to immunotherapy. As immunotherapy is used increasingly in this setting, future directions for research may include examining the impact of its combination with radiation on osseous disease.

## 5. Conclusions

In summary, despite previous in vitro studies demonstrating radioresistance of RCC, there have been numerous clinical studies displaying the efficacy of radiation in the management of osseous metastases from this disease. Conventionally fractionated radiation therapy remains an appropriate option for management of these patients. Given the equivalent pain control rates but inferior local control rates of conventional fractionation when compared to SBRT, clinical judgment may play an important role in appropriate patient selection for these different treatment modalities. SBRT may be preferred in patients with longer life expectancy and good performance status, with lesions in weight-bearing bones, and with nonclear cell histologies. Further study is warranted to identify patients in whom the improved disease control provided by SBRT would be most appropriate.

## Figures and Tables

**Table 1:** Characteristics of patients and individual metastases.

Characteristic	Number of patients (%)

Total	40 (100%)
Gender	
Male	31 (77.5%)
Female	9 (22.5%)
Median age	63 (range, 39–83 years)
Median follow-up	9.3 (range, 1.6–71.5 months)

Characteristic	Number of metastases (%)

Total	53 (100%)
Radiation therapy course	
30 Gy in 10 Fx	16 (30.2%)
20 Gy in 5 Fx	7 (13.2%)
39 Gy in 13 Fx	7 (13.2%)
30 Gy in 5 Fx	5 (9.4%)
25 Gy in 5 Fx	3 (5.6%)
37.5 Gy in 15 Fx	3 (5.6%)
36 Gy in 12 Fx	2 (3.8%)
39 Gy in 15 Fx	2 (3.8%)
8 Gy in 1 Fx	1 (1.9%)
12 Gy in 2 Fx	1 (1.9%)
27 Gy in 8 Fx	1 (1.9%)
30 Gy in 12 Fx	1 (1.9%)
35 Gy in 14 Fx	1 (1.9%)
36 Gy in 6 Fx	1 (1.9%)
45 Gy in 9 Fx	1 (1.9%)
45 Gy in 15 Fx	1 (1.9%)
Median BED	43 (range, 17–74 Gy)
Surgery	
Yes	20 (37.6%)
No	33 (60.4%)
Site	
Vertebra	23 (43.4%)
Long bones	8 (15.1%)
Pelvis	12 (22.6%)
Others^*∗*^	10 (18.9%)
Concurrent treatment	
Yes	25 (47.1%)
No	27 (50.1%)
Clear cell histology	
Yes	44 (83.0%)
No	9 (17.0%)

^*∗*^Other sites include ribs, clavicle, and clivus; Gy indicates Gray; Fx indicates fractions; BED indicates biological equivalent dose.

**Table 2:** The effect of patient characteristics and treatment factors on outcomes.

Covariate	Initial pain response *p* value	Pain recurrence *p* value	Radiographic recurrence *p* value
Age	**0.02** ^*∗*^	0.32	0.95
Gender	0.51	0.47	0.23
Surgery	0.92	0.60	0.63
Concurrent treatment	0.65	0.15	0.24
Clear cell histology	0.75	**0.007** ^*∗*^	0.25
Site	0.77	0.87	0.76
BED	0.46	0.10	0.35
Total dose	0.08	0.20	0.23
Initial pain response	N/A	N/A	**0.032** ^*∗*^

Logistic regression was used for univariate analyses. BED indicates biological equivalent dose. ^*∗*^Denotes significant *p* value (*p* < 0.05).

## Abbreviations

RCC: Renal cell carcinoma
SBRT: Stereotactic body radiation therapy
RT: Radiation therapy
TKI: Tyrosine kinase inhibitor
Gy: Gray
BED: Biological effective dose.
